# Does a 6-Month Multicomponent Training Improve Functional Capacity of Individuals With a Neurocognitive Disorder?

**DOI:** 10.1093/geroni/igab046.3247

**Published:** 2021-12-17

**Authors:** Oscar Ribeiro, Flávia Borges-Machado, Duarte Barros, Laetitia Teixeira, Joana Carvalho

**Affiliations:** 1 University of Aveiro, University of Aveiro, Aveiro, Portugal; 2 Research Centre in Physical Activity, Health and Leisure (CIAFEL), Faculty of Sports, University of Porto, University of Porto, Porto, Portugal; 3 ICBAS.UP, ICBAS.UP, Porto, Portugal

## Abstract

Regular physical activity and exercise have been proposed as non-pharmacological therapeutic approaches to prevent and manage neurocognitive disorders (NCD). Multicomponent training (MT) combining aerobics, strength, postural and balance exercises seem to be effective at improving individuals with NCD in their ability to independently perform activities of daily living (ADL). This quasi-experimental controlled trial aims to analyze the effects of a 6-month MT intervention on functional capacity of individuals diagnosed with NCD. Forty-three subjects (N Major NCD: 36) participated in the Body&Brain Project and were subdivided in exercise group (EG; N: 23; 75.09 ± 5.65 years; age range: 61-83) or a control group (CG; N:20; 81.90 ± 5.95 years; age range: 70-89). The EG was submitted to bi-weekly exercise sessions, and the CG received monthly recreation sessions. At baseline and at post-intervention Timed-Up-and-Go (TUG), 6-meters Walk Speed and Handgrip tests were applied to evaluate lower body mobility, walking speed and handgrip strength, respectively. Results from linear mixed models revealed a statistically significant interaction between group (intervention vs. control) and time for TUG and walk speed test, but not for handgrip strength. The 6-month MT intervention improved lower body mobility and walking speed of older adults diagnosed with NCD, which might potentially impact ADL independence and quality of life. Trial registration: ClinicalTrials.gov - NCT04095962. Supported by FCT: “Body and Brain” (POCI-01-0145-FEDER-031808), CIAFEL (FCT/UIDB/00617/2020), and Ph.D. Grants (SFRH/BD/136635/2018) to FM and [2020.05911.BD] to DB.

